# Ethyl Pyruvate Emerges as a Safe and Fast Acting Agent against *Trypanosoma brucei* by Targeting Pyruvate Kinase Activity

**DOI:** 10.1371/journal.pone.0137353

**Published:** 2015-09-04

**Authors:** Netsanet Worku, August Stich, Arwid Daugschies, Iris Wenzel, Randy Kurz, Rene Thieme, Susanne Kurz, Gerd Birkenmeier

**Affiliations:** 1 Institute of Biochemistry, Faculty of Medicine, University of Leipzig, Johannisallee 30, 04103, Leipzig, Germany; 2 Department of Tropical Medicine, Medical Mission Institute, Salvatorstrasse 7, 97067, Würzburg, Germany; 3 Department of Veterinary Parasitology, Faculty of Veterinary Medicine, University of Leipzig, An den Tierkliniken 35, 04103, Leipzig, Germany; 4 Department of Physics, Faculty of Natural Sciences, University of Leipzig, Linnéstrasse 5, 04103, Leipzig, Germany; 5 Department of Visceral, Transplantation, Thoracic and Vascular Surgery, University Medical Center Leipzig, Liebigstrasse 20, 04103, Leipzig, Germany; University of Hull, UNITED KINGDOM

## Abstract

**Background:**

Human African Trypanosomiasis (HAT) also called sleeping sickness is an infectious disease in humans caused by an extracellular protozoan parasite. The disease, if left untreated, results in 100% mortality. Currently available drugs are full of severe drawbacks and fail to escape the fast development of trypanosoma resistance. Due to similarities in cell metabolism between cancerous tumors and trypanosoma cells, some of the current registered drugs against HAT have also been tested in cancer chemotherapy. Here we demonstrate for the first time that the simple ester, ethyl pyruvate, comprises such properties.

**Results:**

The current study covers the efficacy and corresponding target evaluation of ethyl pyruvate on *T*. *brucei* cell lines using a combination of biochemical techniques including cell proliferation assays, enzyme kinetics, phasecontrast microscopic video imaging and *ex vivo* toxicity tests. We have shown that ethyl pyruvate effectively kills trypanosomes most probably by net ATP depletion through inhibition of pyruvate kinase (K*i* = 3.0±0.29 mM). The potential of ethyl pyruvate as a trypanocidal compound is also strengthened by its fast acting property, killing cells within three hours post exposure. This has been demonstrated using video imaging of live cells as well as concentration and time dependency experiments. Most importantly, ethyl pyruvate produces minimal side effects in human red cells and is known to easily cross the blood-brain-barrier. This makes it a promising candidate for effective treatment of the two clinical stages of sleeping sickness. Trypanosome drug-resistance tests indicate irreversible cell death and a low incidence of resistance development under experimental conditions.

**Conclusion:**

Our results present ethyl pyruvate as a safe and fast acting trypanocidal compound and show that it inhibits the enzyme pyruvate kinase. Competitive inhibition of this enzyme was found to cause ATP depletion and cell death. Due to its ability to easily cross the blood-brain-barrier, ethyl pyruvate could be considered as new candidate agent to treat the hemolymphatic as well as neurological stages of sleeping sickness.

## Introduction

Human African Trypanosomiasis (HAT) also called sleeping sickness is a re-emergent disease, but does not attract much attention, probably because its impact is regional.

The two subspecies of *T*. *brucei* known to cause HAT are *T*. *brucei gambiense (T*. *b*. *gambiense)* and *T*. *brucei rhodesiense (T*. *b*. *rhodesiense)*. A third subspecies, *T*. *brucei brucei (T*. *b*. *brucei)*, is only infectious to animals and is commonly used as an experimental model for the two other parasite species [1,2,3]. The *T*. *b*. *gambiense* brings the chronic form of HAT in West and Central Africa, representing more than 98% of all reported cases. *T*. *b*. *rhodesiense* results in the acute form of the disease in East and Southern Africa, representing 2% of all reported cases. Until now, it has been estimated that the actual number of cases is at least 20,000–30,000 the vast majority of which are not diagnosed or treated [4].

Currently available drugs suffer from contraindications; meanwhile, patient demands for hospitalization and treatment is high [5,6,7]. Identification of innovative drug targets that are safe, efficacious, cost effective and easy to administer is therefore a research priority.

A new treatment option, nifurtimox-eflornithine combination therapy (NECT), recently listed under the essential drugs of WHO to treat neglected tropical diseases, seems promising except that it still produces side-effects in 68% of patients [8,9].

Some trypanocidal drugs have been previously investigated for their anti-cancer activities [10]. The probable similarities among trypanosome and cancer cells are their fast proliferation characteristics and their robust glycolytic pathway [11,12]. However, the glycolytic chain in *T*. *brucei* has a number of peculiarities. Most of its glycolytic enzymes are localized within membrane bound organelles called glycosomes and pyruvate is released out of the cells as a final product of the glycolysis instead of lactate in mammalian cells. *T*. *brucei* bloodstream forms essentially depend on glycolysis as many enzymes of the tricarboxylic acid cycle and cytochromes are not expressed in the mitochondrion [13]. This shows the physiological essentiality of pyruvate export in *T*. *brucei*. A recent study has also confirmed the role of pyruvate transporter blockage as a promising target in trypanosomes [14]. Recently, the dicarbonyl compound, ethyl pyruvate, has been shown to combat glycolysis-driven tumor cells through inhibition of the activity of glyoxalases, which are involved in metabolism of methyglyoxal [15,16].

However, this mechanism may not be the same in *T*. *brucei* cells because of the absence of a functional glyoxalase I enzyme [17]. Besides the speculated mechanisms of action of ethyl pyruvate at the cellular level e.g. inhibition of the NF-kappaB pathway may not apply for *T*. *brucei* [18].

Surprisingly, in the present study we observed a strong anti-trypanosomal activity of ethyl pyruvate. Our current results indicate that this is most probably brought about by inhibition of an important regulatory enzyme within the glycolytic pathway, namely pyruvate kinase. Inhibition of this enzyme was found to cause a significant and fast depletion of ATP and consequently death of trypanosome cells. These results indicate that ethyl pyruvate might be a lead compound which warrants further structural optimization to become a promising candidate for the treatment of trypanosomiasis.

## Results

### Effect of ethyl pyruvate on *T*. *brucei* cells proliferation and their energy metabolism

**Fig 1:**
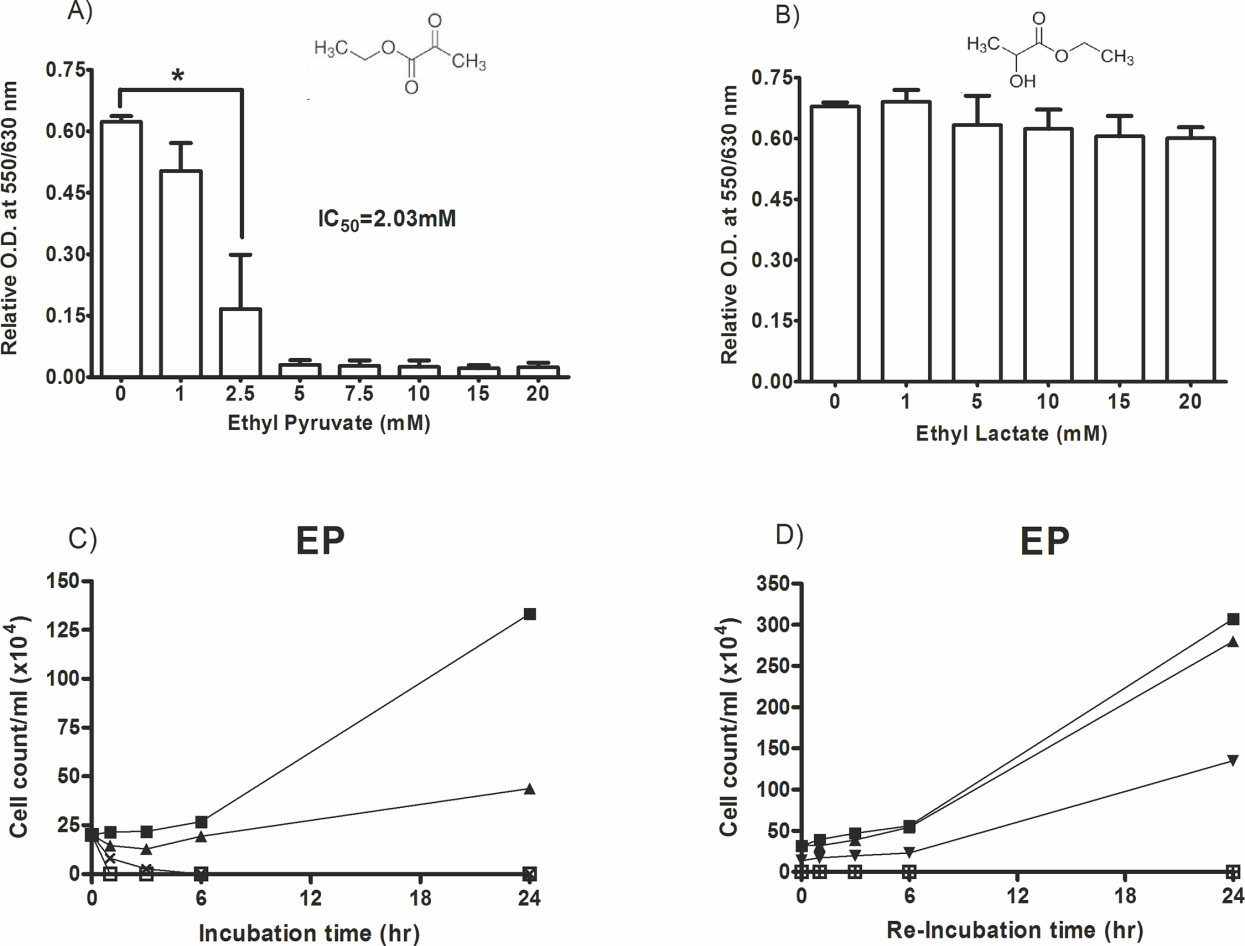
Effect of ethyl pyruvate and ethyl lactate on T. brucei cells proliferation. A cell proliferation/viability assay was conducted in 96-well cell culture plates containing 2x104 cells, medium and ethyl pyruvate in a volume of 200 μl. After 24 hrs of incubation the AlamarBlue cell proliferation reagent (20 μl) was added and the absorbance was read: (A) Ethyl pyruvate; (B) Ethyl lactate; (C) For the time-dependency test 2x105 cells/ml were cultured in 24-well plates in the presence of different concentrations of ethyl pyruvate at 0 mM (■), 1 mM (▲), 2.5 mM (x) and 5 mM (□). After certain time intervals aliquots were removed and the number of vital cells was counted. (D) For cell-recovery test 2x105 cells/ml were cultured in the presence of ethyl pyruvate at 0 mM (■), 1 mM (▲), 2.5 mM (▼), 5 mM (□) for 3 hrs in 24-well plates. Then, the medium was removed and the cells were replenished with fresh medium without the ethyl pyruvate and further cultured for 24 hrs. At the indicated time points aliquots were removed and vital cells were counted. Cells were cultured at 37°C containing 5% CO2 in a 100% humidified environment, * p<0.05.

We first analysed the effect of increasing concentrations (1–20 mM) of ethyl pyruvate and its reduced form, ethyl lactate, on proliferation of the *T*. *brucei* cells (strain TC-221). The samples without ethyl pyruvate were considered as blank controls. The result revealed that ethyl pyruvate effectively inhibits cell proliferation with an IC_50_ of 2.03 ±0.25 mM. The minimum ethyl pyruvate concentration that gives maximum inhibition was 5 mM (Fig 1A). Interestingly, ethyl lactate did not inhibit the cells (Fig 1B). This was surprising, because the difference between the two compounds is just the presence of two additional protons in ethyl lactate.

Next we analysed the effect of ethyl pyruvate at different time points of incubation. The proliferation of trypanosomes was completely inhibited at 5 mM ethyl pyruvate after 3 hrs of incubation indicating that ethyl pyruvate kills trypanosomes within a short time of contact. At lower concentrations of ethyl pyruvate the inhibitory effect was reduced. No inhibition was observed at concentrations <1 mM ethyl pyruvate (Fig 1C).

To analyse whether ethyl pyruvate inhibits trypanosomes irreversibly, trypanosomes were incubated with variable concentrations of ethyl pyruvate for 3 hrs, then the medium was removed and the cells were finally replenished in fresh medium in the absence of ethyl pyruvate for 24 hrs (Fig 1D). Cells exposed to low concentration of the ethyl pyruvate (≤ 2.5 mM) preserved their vitality, continued to proliferate and were capable of recovering from the cytotoxic effect of ethyl pyruvate. However, at a concentration of ≥5 mM the cells were irreversibly damaged and completely lost their cellular integrity.

### *In vitro* drug resistance

**Fig 2:**
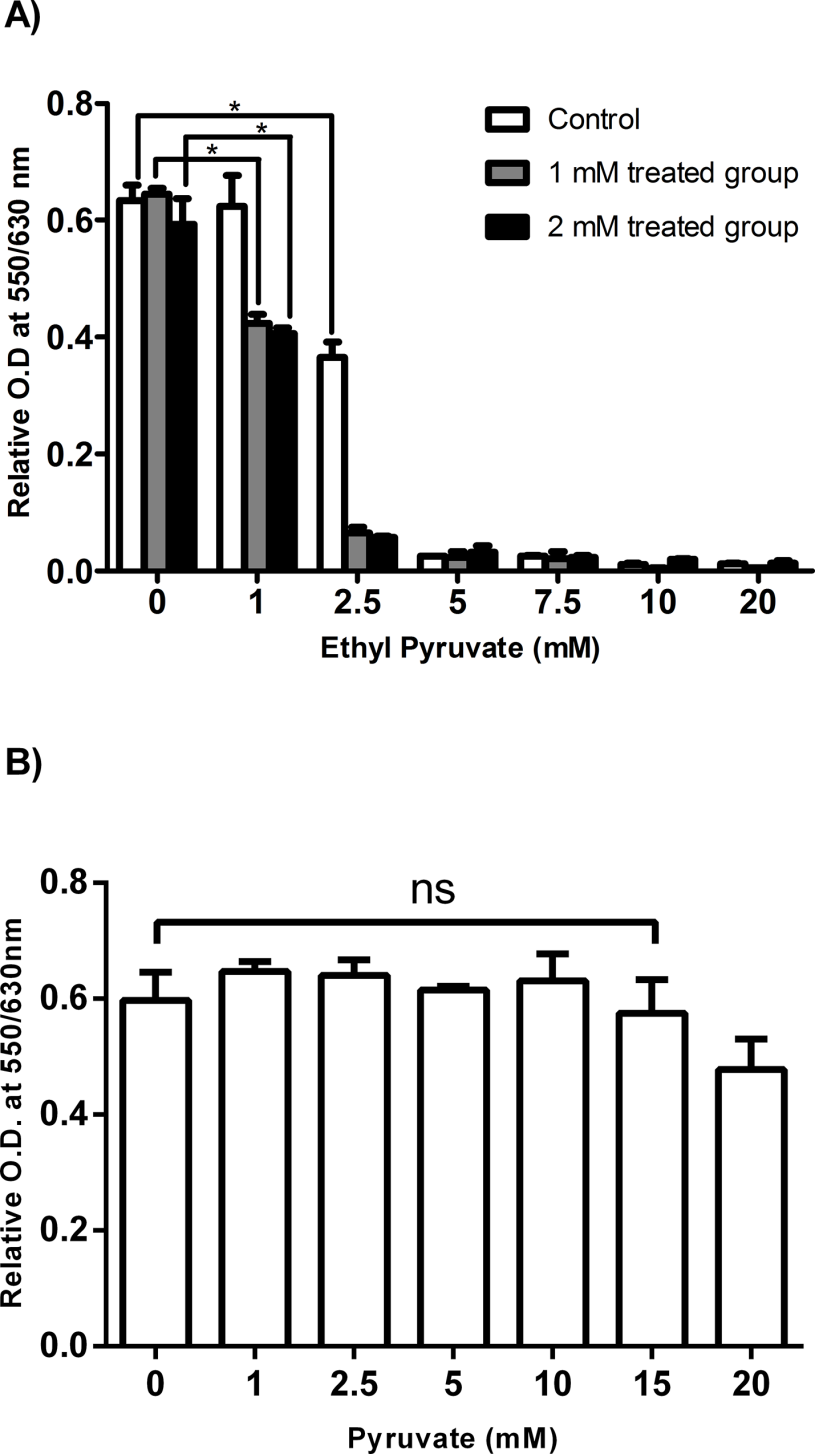
Drug resistance test for *T*. *brucei* using sublethal concentrations of ethyl pyruvate and effect of pyruvate on cell proliferation. (A) Cells were treated with sub-lethal concentration of ethyl pyruvate, 1 mM and 2 mM, respectively, for 30 days in separate cell culture flasks. The medium and the trypanocidal compound were changed every two days. The control cells were left in fresh medium without ethyl pyruvate. On day 31, 2x104 cells/100μl from each flask were suspended a 96-well plate. Each cell group was treated with ethyl pyruvate at increasing concentrations ranging from 1 to 20 mM. Absorbance was recorded at 48 hrs of incubation. (B) The impact of pyruvate (0–20 mM) on cell proliferation was analysed as described in Fig 1.

The rationale of this test was to analyse whether the trypanosomes become resistant to treatment by ethyl pyruvate. Therefore, *T*. *brucei* parasites were cultured in medium containing sub-lethal concentrations of ethyl pyruvate of 1 mM and 2 mM, respectively, for 30 days accompanied by changing of the medium every two days. Then, the cells were subjected to increasing concentrations of ethyl pyruvate (1 to 20 mM) and the proliferation capacity was determined. The results finally showed that the parasites failed to develop resistance against ethyl pyruvate during the selected period of exposure at least under *in vitro* conditions (Fig 2A). In addition to ethyl pyruvate and ethyl lactate, the sodium salt of pyruvate was also tested for its effect on *T*. *brucei* cells proliferation. The result showed no statistically significant inhibition at least in the range from 0 to 15 mM (Fig 2B).

### Comparative anti-proliferative effects of pentamidine and suramin

**Fig 3:**
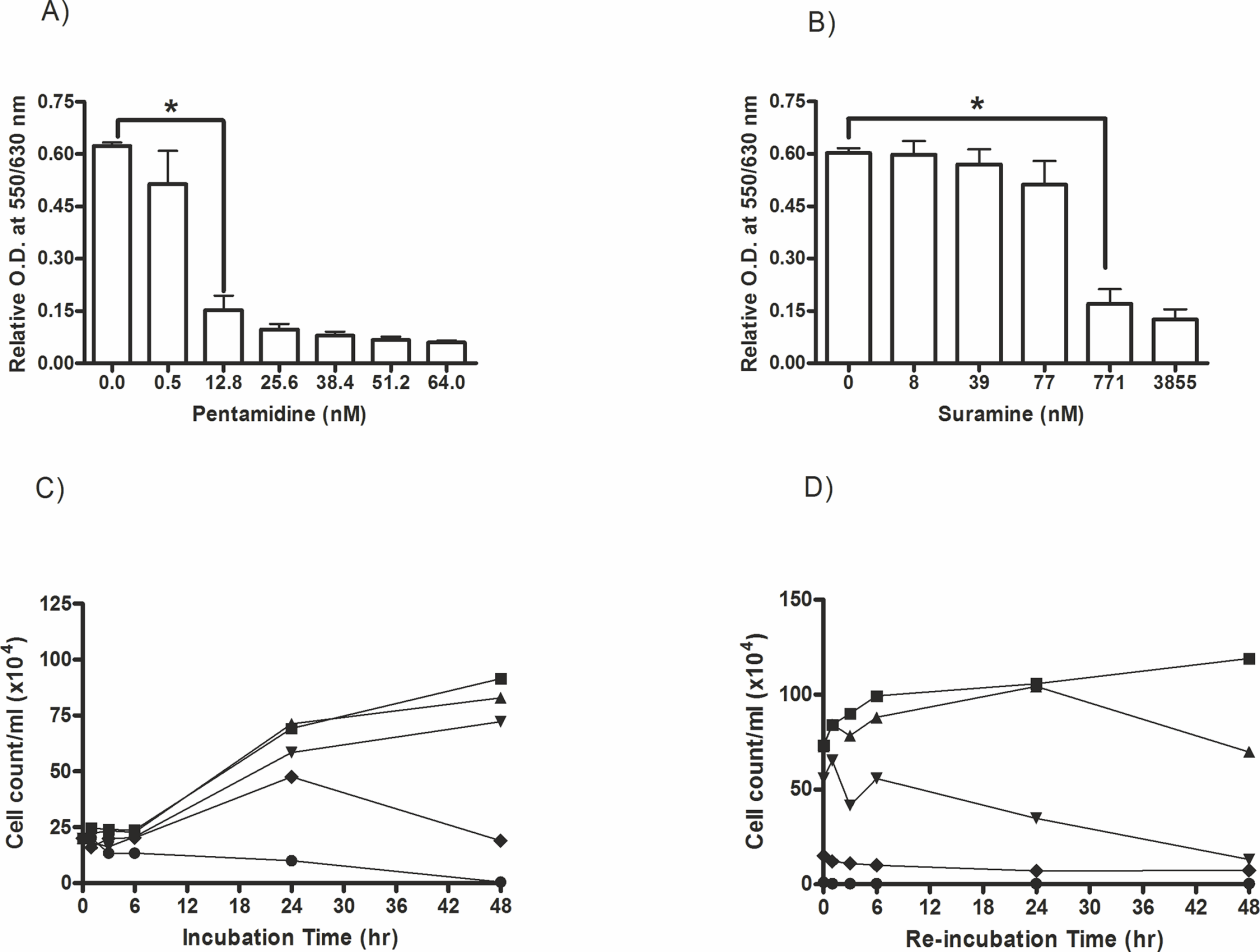
Anti-proliferative effects of pentamidine and suramin on *T*. *brucei*. Cell proliferation/viability was assayed as described in Fig 1 with the exception of the use of (A) pentamidine and (B) suramin at variable concentrations. (C) Time dependency of anti-proliferative effects of pentamidine at variable concentrations (12.8 to 128 nM) was performed analogously to the experiments shown in Fig 1. Control samples contained 100 μl of fresh medium instead of a trypanocidal compound. Pentamidine concentrations used were: 0 nM (■), 12.8 nM (▲), 51.2 nM (▼), 64 nM (♦) and 128 nM (●). (D) Recovery test of *T*. *brucei* cells exposed to pentamidine was done in 24-well in the presence of pentamidine (12.8 nM to 128 nM). Pentamidine concentrations used were: 0 nM (■), 12.8 nM (▲), 51.2 nM (▼), 64 nM (♦) and 128 nM (●).The results depict the mean of three independent measurements (n = 3), *p<0.05.

Currently, the registered first-stage sleeping sickness treatment drugs are pentamidine and suramin. Suramin is an analogue of Trypan Blue that has been used for treatment of sleeping sickness for many years [19]. Pentamidine belongs to the class of aromatic diamidines previously designed for prevention and treatment of pneumocystic pneumonia, leishmaniasis and first stage infection of *T*. *gambiense* [20]. It was our intent to compare the anti-trypanosomal activity of ethyl pyruvate with these standard drugs used in sleeping sickness treatment. As demonstrated in Fig 3A the proliferation of trypanosomes is inhibited by pentamidine at quite lower concentrations compared to ethyl pyruvate. Suramin demonstrated anti-proliferative effects in the range of 100 nM and 1 μM, however no complete inhibition even after 48 hrs of incubation at the maximum applied concentration of 3855 nM was achieved (Fig 3B). The time-dependency test revealed measurable effects of pentamidine only at 48 hrs of incubation (Fig 3C). The death rate of pentamdine treated *T*. *brucei* indicated that more than 80% of the cells could be killed after 24 hrs of incubation and complete inhibition of cell proliferation was obtained only at the highest concentration of 128 nM and after 48 hrs. The cell-recovery tests with pentamidine confirmed its cytostatic effect at 64 nM and full cytotoxicity (non-recovery) at concentrations ≥128 nM at long term incubation (Fig 3D). Compared to pentamidine, ethyl pyruvate, however, was a very fast acting and irreversible growth inhibitor.

### Effect of ethyl pyruvate at cellular ATP level

**Fig 4:**
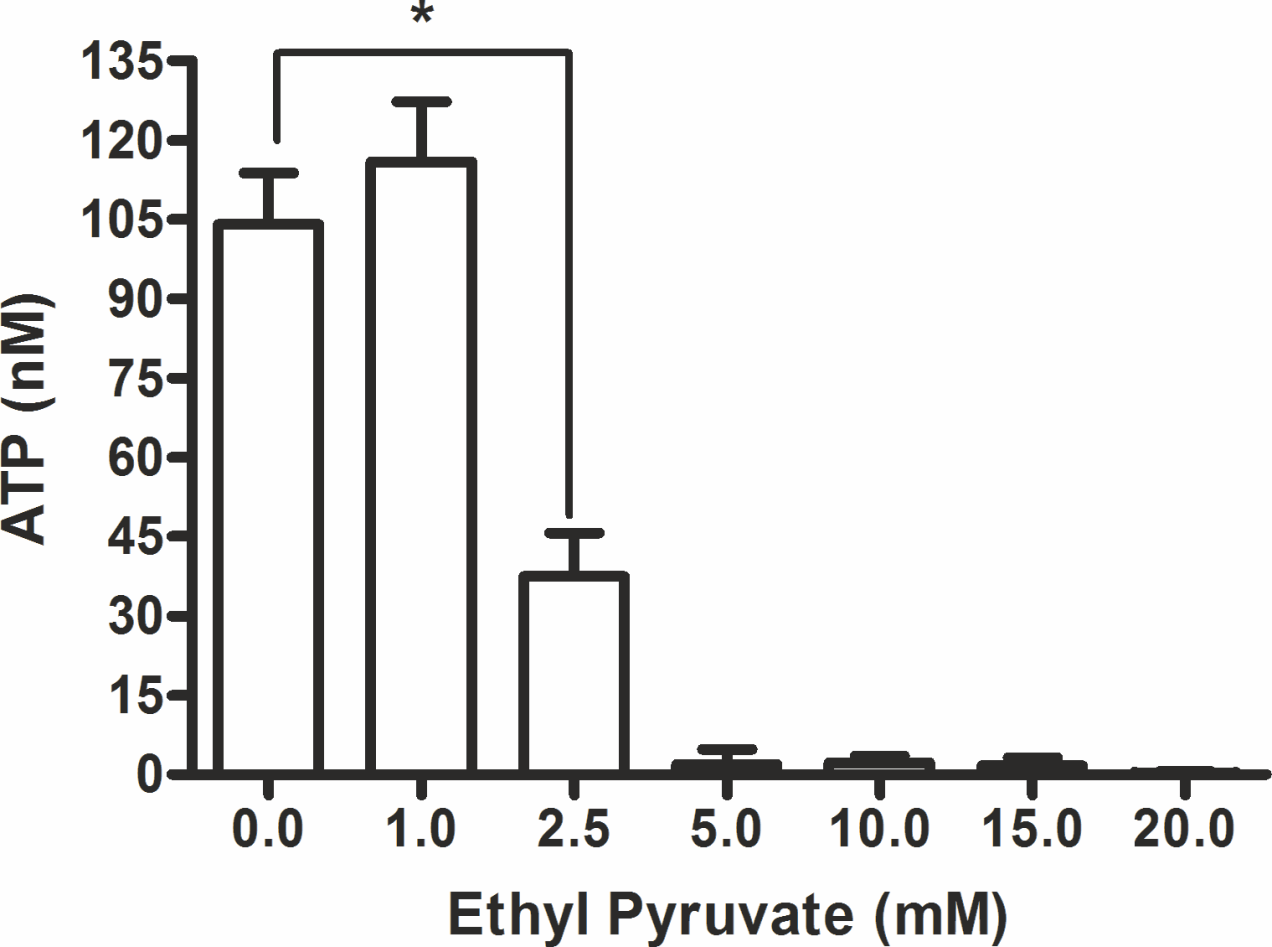
Effect of ethyl pyruvate at cellular ATP level in *T*. *brucei*. Trypanomomes (3x10^5^ cells/well) were cultured in 24-well plates in the absence (control) or presence of ethyl pyruvate (1 to 20 mM) and incubated for 3 hrs at 37°C in 5% CO_2._ After incubation, cells were centrifuged at 1500 rpm for 10 min and supernatant was removed. The cell sediment was re-suspended smoothly in 500 μl of fresh medium. From this suspension 50 μl (equivalent to 3x10^4^ cells) was added into a 96-well black opaque-walled plate in duplicates and mixed with 50 μl of test reagent per well. Luminescence was read at 560 nm. The actual ATP level was calculated from the trend line formula of the standard curve (n = 3), *p<0.05.

The sudden loss of viability of trypanosomes at short-time incubation with ethyl pyruvate prompted us to look at the energy budget of the cells assuming that ATP depletion may be a possible cause of its extreme cytotoxicity against *T*. *brucei*. Our results revealed that the cellular ATP level was significantly hampered. There was a clear concentration-dependent loss of cellular ATP in the presence of ethyl pyruvate (Fig 4). The result indirectly demonstrated that ethyl pyruvate might hamper the glycolytic pathway which is the sole energy-rich phosphate delivering pathway in trypanosomes.

### Effect of ethyl pyruvate on the activity of glycolytic enzymes

**Fig 5:**
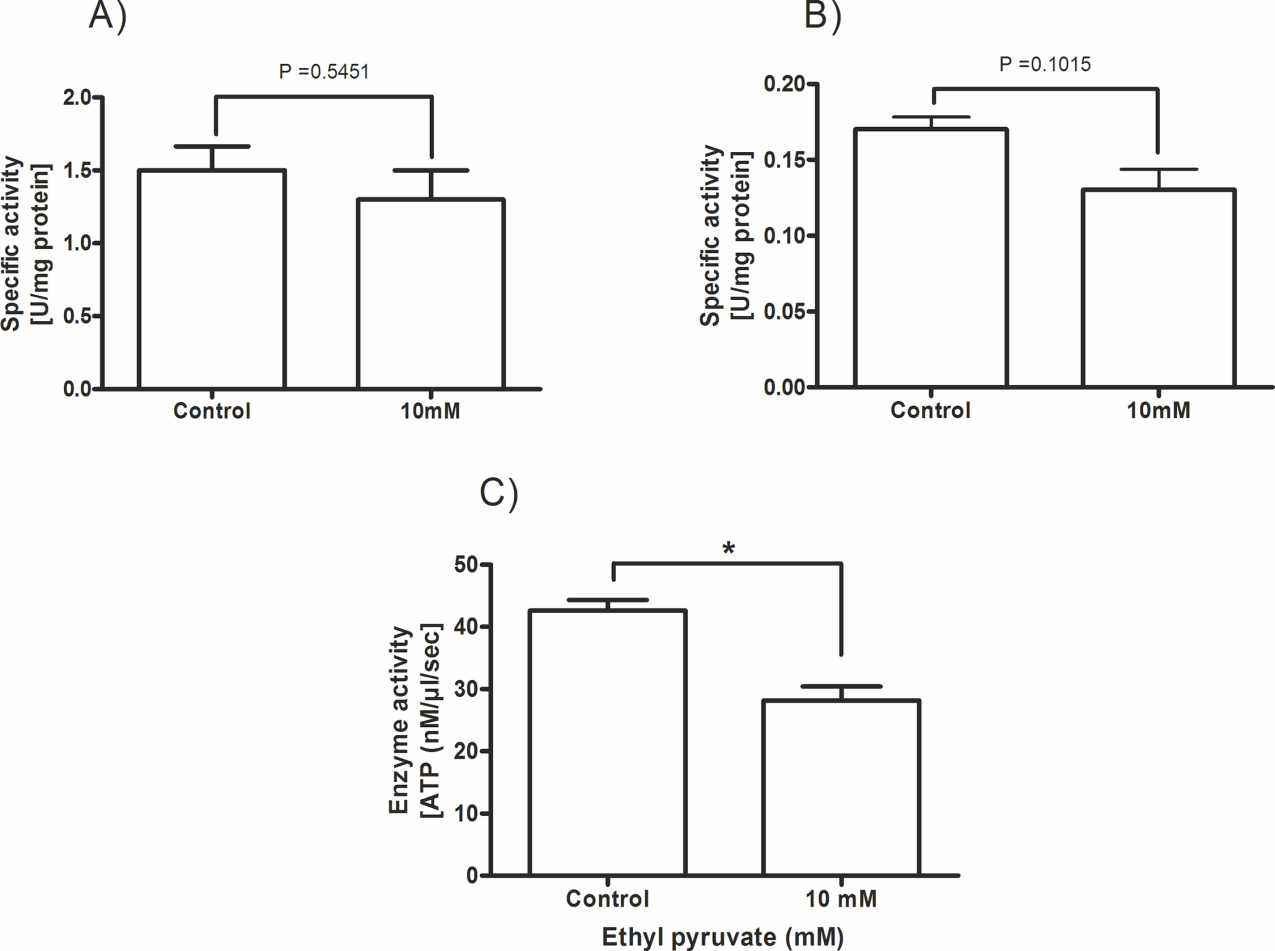
Effect of ethyl pyruvate on the activity of glycolytic enyzmes of *T*. *brucei*. Specific activity of glycolytic enzymes in *T*. *brucei* cell extracts in the absence and presence of 10 mM ethyl pyruvate. (A) hexokinase, (B) phosphofructokinase and (C) pyruvate kinase. All cell extracts were pre-treated with ethyl pyruvate for 30 min before activity testing. All experiments were performed in triplicates (n = 3), * p<0.05.

The results shown above let us assume that ethyl pyruvate most probably interferes with the glycolytic pathway in trypanosomes. The enzymes critical for the survival of blood stream form trypanosomes that might directly affect the ATP production are hexokinase-1 (HK-1), phosphofructokinase (PFK) and pyruvate kinase (PK). Therefore, we analyzed the specific activity of these glycolytic enzymes in trypanosome extracts in the absence and presence of 10 mM ethyl pyruvate. This concentration was selected because of its maximum effects obtained in all tests so far. As demonstrated in Fig 5A and 5B, ethyl pyruvate was unable to inhibit the activity of HK-1 and PFK in cell extracts. In contrast to these enzymes the activity of the PK was significantly inhibited at a concentration of 10 mM ethyl pyruvate (Fig 5C).

**Fig 6:**
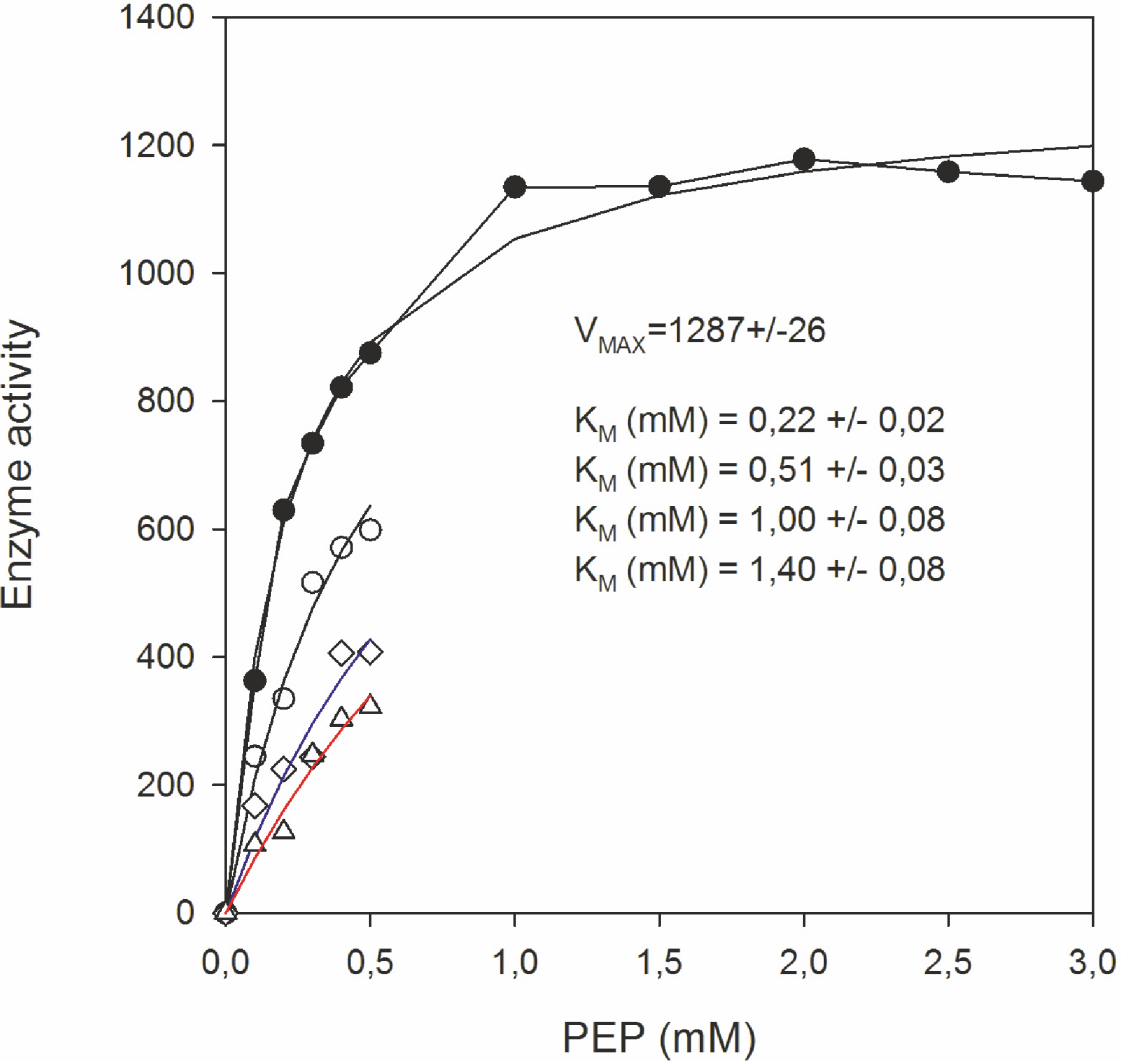
Competitive inhibition of pyruvate kinase activity by ethyl pyruvate. Enzyme activity was measured by following the production of ATP from the substrates phosphoenolpyruvate (PEP) and ADP as described in Materials and Methods. Enzyme activity expressed as nmole ATP/μl/s (ordinate) was recorded in dependence of increasing substrate concentrations (PEP) in the absence and presence of ethyl pyruvate. Controls were (●) without ethyl pyruvate (K_*m*_ = 0.22±0.02 mM); (○) 5 mM ethyl pyruvate (K_*m*_ = 0.51±0.03mM); (◊) 10 mM ethyl pyruvate (K_*m*_ = 1±0.08mM) and (∆) 15 mM ethyl pyruvate (K_*m*_ = 1.4±0.08mM). The pure enzyme from rabbit muscle (200 U/mg) was used for the experimental setup to avoid contamination bias in our cell extract. The K*m* and K*i* values were calculated by SIGMAPLOT (Systat Softwarwe Inc.).

In order to exclude a possible influence of cellular metabolites of the extract on PK kinetics we used purified PK from rabbit muscle for detailed kinetic studies. Thus, we analyzed the substrate-enzyme activity relation in the absence and presence of variable concentrations of ethyl pyruvate and phosphoenolpyruvate (PEP), respectively. Fig 6 shows the K*m* (± SD) values for the control (without ethyl pyruvate), and at different ethyl pyruvate concentrations in dependence of the substrate concentration. From the obtained saturation curves the K*i* value was calculated. Data analysis revealed a competitive inhibition of PK activity by ethyl pyruvate with a K_*i*_ = 3.0±0.29 mM.

### Cytotoxicity of ethyl pyruvate to human red blood cells

**Fig 7:**
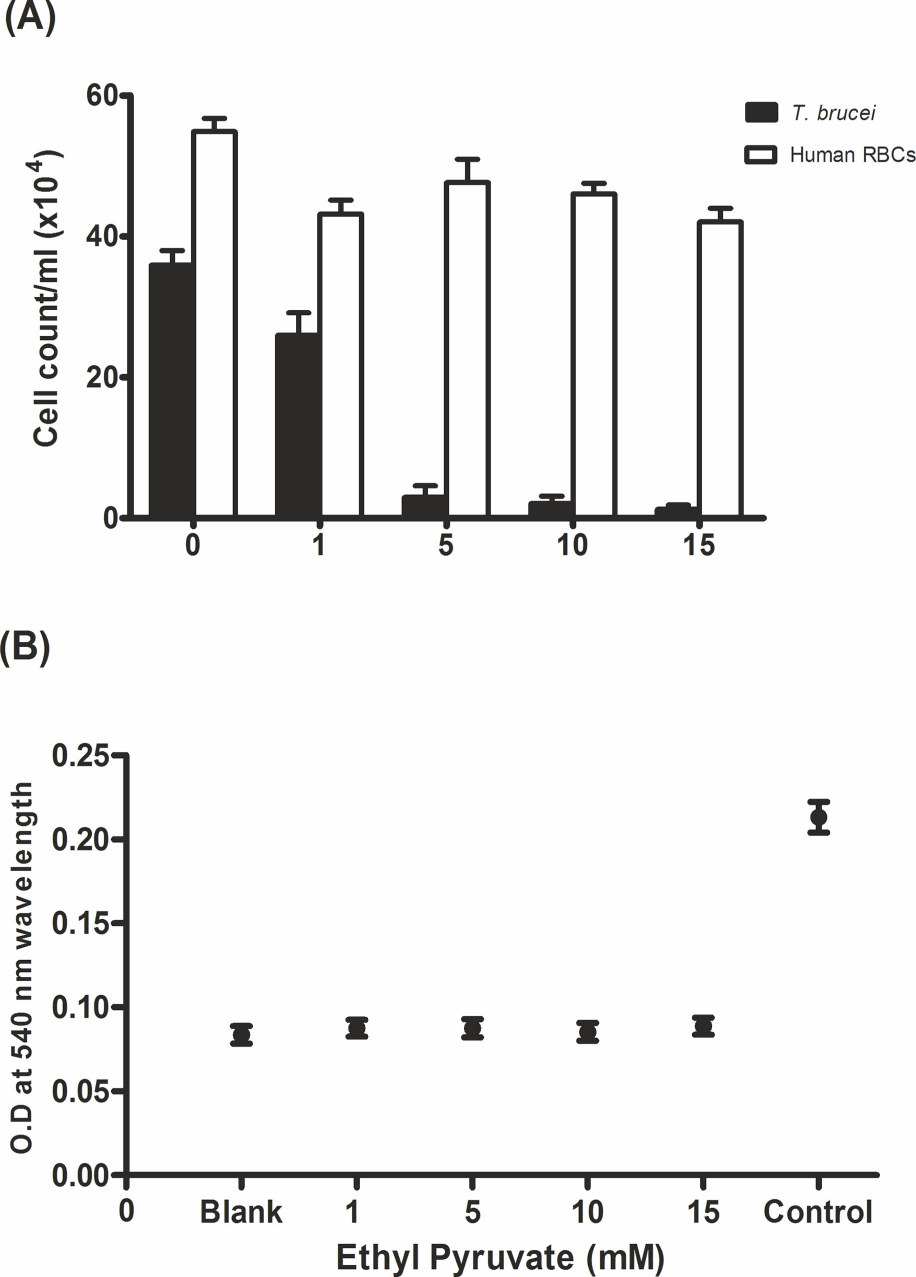
Cytotoxicity evaluation of ethyl pyruvate on human erythrocytes. (A) 6x10^5^ red blood cells were mixed to 2x10^5^ *T*. *brucei* cells in 5 ml fresh medium in a flask. Cells were first incubated at 37°C in 5% CO_2_ for 24 hrs. Out of this 1 ml-aliquots were distributed into 24-well plates and incubated with 100 μl of increasing concentrations of ethyl pyruvate (1–15 mM). Controls contained cells without ethyl pyruvate but 100 μl of fresh medium. The cells were then re-incubated for 3 hrs. Afterwards, the number of trypanosomes and human erythrocytes was counted using a haemocytometer. (B) Effect of ethyl pyruvate (10 mM) on red blood cell haemolysis was determined by measuring the optical density of the medium containing the *T*. *brucei* and red blood cells co-incubated for 3 hrs. Before measurement, the cells were spun down by centrifugation. Total haemolysis of an equivalent number of cells (labelled as ‘Control’) was achieved by sonication (70% power, 5x5 sec) using an ultrasonicater. Cells without ethyl pyruvate were used as negative controls (labelled as ‘Blank’).

To prove whether ethyl pyruvate displayed advantageous selectivity of action against protozoa cells we performed *ex vivo* experiments by co-incubation of human red blood cells with the trypanosomes and ethyl pyruvate for 3 hrs, followed by cell counting and analysis of the red blood cell hemolysis. As expected, at 5 mM ethyl pyruvate only a few motile cells were observed while no significant change was seen in the red blood cell count (Fig 7A). The latter finding was substantiated by a non-significant release of hemoglobin from the red blood cells that remarks the ethyl pyruvate´s safety at least toward human erythrocytes when compared with the OD of totally haemolysed blood cells of the same sample used as a positive control (Fig 7B).

### Phase contrast microscope video imaging of ethyl pyruvate exposed *T*. *brucei* cells

To visualize the effectiveness and fast acting property of ethyl pyruvate we performed video-recording and phase contrast microscopy of the trypanosome.

The videos showed the features of morphological changes of trypanosoma cells throughout the 3 hrs incubation time. The cell motility status generally indicates how fast ethyl pyruvate acts in killing these cells. The fast acting property of ethyl pyruvate can easily be confirmed by the slow or static motion of the previously active motile cells. The cytotoxic effect of ethyl pyruvate started already after 1 hr of exposure and complete inhibition of cell motility finally leading to cell death was observed after 3 hrs when incubated with 5 mM of the compound. In contrast to ethyl pyruvate, no visible effect was displayed when using pentamidine at the highest toxic concentration of 128 nM within the same time range. The videos show the control cells without treatment (S1 Video), cells treated with 128 nM pentamidine (S2 Video) and cells treated with 5 mM ethyl pyruvate (S3 Video).

## Discussion

Even though the trypanosome genome was sequenced many years ago, no new breakthroughs in the treatment of HAT have yet emerged [21], despite the fact that some promising compounds are now in the development pipeline [22,23]. Notably, drugs such as suramin and nifurtimox with anti-trypanosomal activity have been tested in cancer therapy, too [24,25,26].

A common feature between tumor cells and trypanosomes is the high consumption of glucose. To cover its high energy demand, the parasite uses the host’s glucose for ATP production [27,28]. Within the glycolytic chain phosphoenolpyruvate is finally converted to pyruvate and ATP by cytosolic pyruvate kinase. Pyruvate is then released from the cell because of the lack of a canonical L-lactate dehydrogenase.

Analogously, many tumor cells exhibit a high aerobic glycolysis combined with a restricted oxidation within the respiratory chain [29]. Instead of pyruvate as in trypanonomes, L-lactate is produced by tumor cells which provide a growth advantage because of the repression of the immune system and promotion of cell invasion [30].

Recently, we have shown that compounds containing a dicarbonyl structure such as ethyl pyruvate, curcumin and different polyphenols are capable to kill tumor cells by inhibition of glyoxalases [25,31]. These enzymes prevent glycolysis-driven cellular damage by converting the toxic methyl glyoxal to D-lactate [32].

These findings prompted us to study the effect of ethyl pyruvate on trypanosomes which, like most tumor cells, have a high glycolytic rate. However, we are aware that *T*. *brucei* lacks glyoxalase I and uses trypanothione instead of glutathione unlike in mammalian cells. Nevertheless, we surprisingly found that ethyl pyruvate effectively inhibited proliferation of the trypanosomes.

In search of another target within the glycolytic pathway we analysed key enzymes which are directly involved in the regulation of ATP production in *T*. *brucei*, e.g. HK-1, PFK and PK [33]. While PFK and HK were not inhibited significantly, competitive inhibition of PK (K*i* = 3.0±0.29 mM) by ethyl pyruvate has been ascertained for the first time. PK activity is relatively abundant in the cytosol of *T*. *brucei* and is responsible for the net ATP production by the cell. While most cellular ATP is consumed by hexokinase and phosphofructokinase which are not under regulatory control, inhibition of PK would lead to a rapid loss of cellular ATP. Recently, it was found that loss of PK is lethal to glucose-adapted cells [34]. These data corroborate our results that PK is a new target of ethyl pyruvate. Detailed kinetic analysis revealed a competitive inhibition of PK by ethyl pyruvate. Surprisingly, neither the enzyme nor trypanosome cells could be inhibited by ethyl lactate. The absence of any anti-proliferative effect of this compound suggests that the reduced form is either not taken up by the cells or not converted to ethyl pyruvate because these cells are deprived of lactate dehydrogenase. Admittedly, performing kinetic analysis with mammalian PK and not with purified enzyme from trypanosomes is obviously a drawback of our study. However, trypanosome PK similar to mammalian PK responds to fructose 2,6-bisphosphate as an allosteric activator and require Mg^2+^ ions for full activity [35]. Furthermore, pphylogenetic and structural analysis clearly showed that the enzymes of trypanosomatids and yeast belong within the same grouping of species and demonstrate optimal similarity [36].

We found complete inhibition of trypanosomes at 5 mM ethyl pyruvate within 3 hrs (Fig 1). This effect was irreversible as no recovery of the cells was observed. Although the concentration of ethyl pyruvate (5 mM) is slightly above the K*i*-value of PK (3.0±0.29 mM) for ethyl pyruvate, it in turn seemed to be sufficient to initiate a progressive loss of cellular ATP.

Until recently, the development of drug resistance in microbes and protozoa has been a big challenge in therapeutic medicine. Thus, the lack of drug resistance development we observed in our *in vitro* experiments at least over a time laps of 30 days is very encouraging. This could be due to the attack of more than one target in trypanosomes since it is known that ethyl pyruvate also inhibits glyoxalase II in mammalian as well as yeast cells [15]. This enzyme is present in *T*. *brucei* cells but with yet undefined functions [37]. In contrast to the natural evolutionary pressure in parasites, it is possible that only 30 days of *in vitro* exposure might be too short to draw determinative conclusions. However, we also expect that the laboratory adapted *T*. *brucei* cells are capable of proliferating faster and possibly acquiring drug resistance in a shorter period of exposure.

It has been discussed that monocarboxylates, which are structurally analogous to ethyl pyruvate, affects cell proliferation by blocking the pyruvate transporter. This transporter usually brings the toxic accumulation of the endogenous pyruvate leading to cell death by acidification [38,39,40]. We addressed this question, by exposing trypanosomes with sufficiently higher concentrations of pyruvate. This should stop the efflux of the endogenous pyruvate that might cause the proposed intracellular acidification. However, the impact of extracellular pyruvate on vitality of the *T*. *brucei* cells was negligible (Fig 2B). This may indirectly indicate the requirement of the dicarbonyl structural element in ethyl pyruvate and that pyruvate transporters blockage might still not be the mode of action of ethyl pyruvate in *T*. *brucei* cells [39].

The selective action of ethyl pyruvate was also confirmed by our *ex vivo* investigation showing that human red blood cells were not struck by the compound. This is quite surprising because red blood cells are known to rely on oxidation of glucose to lactate and possess a very active glyoxalase system [41]. In contrast to tumor cells, the lack of damage to red blood cells by the generated methylglyoxal might be due to the absence of mitochondria since methylglyoxal was shown to target mitochondria for induction of cell death and also because of their high level of glutathione [42].

Unlike in *T*. *brucei* the red blood cells are known to release lactate and the *T*. *brucei* pyruvate transporter permeases are not related to MCTs from mammals [43]. These might also be optional reasons why ethyl pyruvate is safe for red blood cells but not to *T*. *brucei*. It should also be considered, that red blood cells might contain a high amount of unspecific esterases which could degrade ethyl pyruvate easily.

Ethyl pyruvate is a comparatively safe and fast acting agent against *T*. *brucei*. However, a relatively high concentration is required to observe optimal inhibition. This might be considered as a constraint when compared with the currently registered drugs acting in nanomolar or micromolar concentrations (Figs 1 and 3). Therefore, for *in vivo* application ethyl pyruvate has to be administered in a concentrated solution by infusion in order to get an effective level of at least 5 millimolar in animals or humans. This route of application was already applied in a phase II clinical study by using 450 mg/kg/day given in five divided doses without side effects [44]. Another possibility of minimizing the required high dose of ethyl pyruvate is to chemically modify the chain length of the alcohol of the ester in order to increase permeability and inhibitory activity. A similar approach was recently demonstrated for optimizing inhibitors for trypanosome PFK and Leishmania PK [45].

## Conclusion

Our *in vitro* experimental study presents ethyl pyruvate as a fast acting, effective and safe trypanocidal compound. Pyruvate kinase has been identified as the new target of ethyl pyruvate. Inhibition of this enzyme was found to cause ATP depletion and sudden cell death. However, further comprehensive studies are needed with respect to *Ki* reduction and toxicity evaluation as well as metabolism in infected animal models. Then, its predefined property to easily cross the blood-brain-barrier could predict its future applicability against both stages of trypanosomiasis [46].

## Materials and Methods

### Blood specimen

Heparinized human blood was obtained from male healthy volunteers in the age of 30 to 40 years. All participants provided their written informed consent to participate in this study. The local ethics committee of the Faculty of Medicine of the University of Leipzig, Germany, approved this study in accordance to the ICH-GCP guidelines (reference number: 057–2010–08032010).

### In vitro cultures of Trypanosoma brucei cells

Laboratory adapted *T*. *b*. *brucei* cells (strain TC-221) were obtained from Prof. A. Stich, Würzburg, Germany [47]. The cells were cultured in complete Baltz medium which was composed of 82% Baltz basic solution, 0.8% ß-mercaptoethanol, 0.8% penicillin/streptomycin (10,000 U/ml), and 16.4% heat inactivated fetal calf serum (FCS). Cells were cultured using 50 ml sterile cell culture flasks (Greiner Bio-One, Frickenhausen, Germany) at 37°C and 5% CO_2_ in 100% humidified environment. The medium was changed every 2–3 days.

### Cell proliferation/viability assay

The AlamarBlue cell proliferation assay (Thermo Fisher Scientific, USA) has been used to measure the metabolically active cells. The principle behind this assay relates to the fact that metabolically active cells create a reducing environment that promotes the conversion of resazurin (non-fluorescent) to resorufin (highly fluorescent). The absorbance was measured using an ELISA reader/96-well multiscanner (Tecan, Crailsheim, Germany) at ex550/em630 nm.

### Concentration-response test

The concentration-response assay was performed in 96-well cell culture plates to which 100 μl *of* a cell suspension (2x10^4^ cells), 100 μl of the trypanocidal compounds at increasing concentrations and 20 μl of AlamarBlue was added. Control wells contained 200 μl of equal number of cells in fresh medium without trypanocidal compounds. The change in absorbance was recorded after 24 hrs of incubation. The drugs or compounds used in different experimental settings were ethyl pyruvate and ethyl lactate, pentamidine-isothionate, suramin and pyruvate (Sigma-Aldrich, Munich, Germany).

### Time-dependency test

The time-dependency test was done in 24-well plates containing 1 ml of cell suspension (2x10^5^ cells/well) and 100 μl of ethyl pyruvate at variable concentrations (1 to 20 mM), or an equal volume of fresh medium (control). Cells were incubated for 24 hrs at 37°C in a humidified environment containing 5% CO_2_. For cell counting, a Neubauer haemocytometer (Marienfeld, Lauda-Königshofen, Germany) was used. The number of moving cells was recorded after 1 hr, 3 hrs, 6 hrs and 24 hrs of incubation, respectively. Tests were done in triplicates and the mean and standard deviation were calculated.

### Cell-recovery test

The cell-recovery test was done using 1 ml of cells (2x10^5^ cells/ml) seeded into a 24-well plate to which was added 100 μl of ethyl pyruvate (1 to 20 mM) or pentamidine (12.8 nM to 128 nM). A volume of 100 μl of fresh medium without trypanocidal compounds served as a control. Cells were exposed to ethyl pyruvate and pentamidine for 3 hrs and 48 hrs, respectively, aliquots were removed and centrifuged (1500 rpm, 10 min, 5°C). The supernatant was carefully removed and the precipitated cells were re-suspended with 1 ml fresh medium without trypanocidal compounds and cultured for another 48 hrs. Vital cells were counted at the beginning of the re-suspension and continued at 1 hr, 3 hrs, 6 hrs, 24 hrs and 48 hrs afterwards. The tests were done in triplicate.

### In vitro drug-resistance test

Primarily, cells were treated with sub-lethal concentrations of ethyl pyruvate (1 and 2 mM) for 30 days in separate cell culture flasks. The medium and the trypanocidal compounds were changed every two days. The control cells were left in fresh medium without ethyl pyruvate. On day 31, 100 μl of 2x10^4^ cells per well were seeded in a 96-well plate. Each cell group was treated with ethyl pyruvate at increasing concentrations ranging from 1 to 20 mM. Controls were incubated in fresh medium without ethyl pyruvate. To each well 20 μl of the AlamarBlue reagent and absorbance was recorded after 48 hrs of incubation.

### Cell extracts preparation and protein concentration determination

Cells were harvested and the pellets were first re-suspended at 1:4 ratio in a lysis buffer containing 25 mM Tris, 2 mM PMSF, 10% Glycerol, 1% Tritonx100, 2 mM EDTA and freshly added 0.3% protease inhibitor cocktail at pH 8.0. The suspension was repeatedly vortexed every 1–2 min within 15 min duration. The cell sediment was always kept on ice. Then the mixture was centrifuged for 15 min at 13000 x *g* and 5°C. The supernatant was carefully pipetted into a new Eppendorf tube and the sediment was discarded. The protein content of samples was determined according to Bradford [48].

### Determination of the ATP content

Cellular ATP content was determined by means of the CellTiter-Glo luminescent cell viability assay according to the manufacturer’s instructions (Promega, Madison, USA). Briefly, cells were cultured in 24-well plates (4x10^4^ cells/well) in the absence or presence of ethyl pyruvate (1 to 20 mM) and incubated at 37°C and 5% CO_2_ for 3 hrs. Cells were then mixed with test reagents and luminescence was read. A standard curve was prepared from ATP (10 nM to 1 μM) in medium to determine the actual ATP level in cells.

### Enzyme activity assays

#### Hexokinase

Hexokinase (HK) (EC 2.7.1.1) activity was measured following up the reduction of NAD^+^ through a coupled reaction with glucose-6-phosphate dehydrogenase. The increase in absorbance was determined spectrophotometrically at 340 nm wavelength according to [49]. Briefly, 100 μl of the cell extract was added to 3 ml of 50 mM Tris-buffer containing 13.3 mM MgCl_2,_ 0.67 M glucose, 16.5 mM ATP, 6.8 mM NAD, pH 8.0, and glucose-6-phosphate dehydrogenase. The measurements were done in the absence and presence of 10 mM ethyl pyruvate, respectively.

#### Phosphofruktokinase

The activity of phosphofructokinase (PFK) (EC 2.7.1.11) in *T*. *brucei* was measured at 340 nm in a coupled assay with aldolase (EC 4.1.2.13), glycerol-3-phosphate dehydrogenase (EC 1.1.1.8) and triosephosphate isomerase (EC 5.3.1.1) according to [50]. Activity of phosphofructokinase was measured using 100 μl of *T*. *brucei* cell extract.

#### Pyruvate kinase

To determine kinetics in pyruvate kinase (PK) (EC 2.7.1.40) activity we first applied the indirect coupled assay using lactate dehydrogenase (LDH) as helper enzyme [49,50]. However, ethyl pyruvate was found to act itself as a substrate of LDH resulting in a concentration-dependent concomitant increase in oxidation of NADH to NAD+. Hence the activity of PK was evaluated by measuring the ATP production using the CellTiter-Glo Luminescent assay following the manufacturer’s instructions (Promega, Madison, USA). To determine the inhibition of PK activity in the cell extracts the reaction was initiated by adding 10 μl of the crude cell extract (1.17 mg/ml protein) to 2 ml of a reaction mixture composed of 200 mM Tris/HCl buffer, 15 mM MgCl_2_, 2 mM ADP, 3 mM phosphoenolpyruvate and 10 mM ethyl pyruvate (pH 7.0). The PK activity was read against a standard curve of ATP and expressed as (nM ATP/μl/s).

The PK kinetics assay was performed by measuring the enzyme activity at increasing concentrations of phosphoenolpyruvate (0.1 to 3 mM) in the absence and presence of ethyl pyruvate (5, 10 and 15 mM). Purified PK-1 from rabbit muscle (stock of 200 U/mg) was used for the test. The activity measurements were performed for 10 min at 20°C. The ATP luminescence measurement was done in a 96-well white opaque plate (Greiner, Frickenhausen, Germany) at 250 milliseconds ATP luminescence integration time using the SoftMax Pro 5.3 software. Finally, the V*max*, K*m* and K*i* values were calculated to characterize the inhibitory effect of ethyl pyruvate at the enzyme. The observed data in the presence and absence of ethyl pyruvate were fit simultaneously for a competitive enzyme inhibition model shown by the equation below. Nonlinear regression fitting was accomplished with use of SIGMAPLOT (Systat Software Inc.) applying a weighting function of 1/Y^2^. PK activity was expressed as (nM ATP/μl/s).

$$V=V_{MAX}*\frac{\left[PEP\right]}{\left([PEP]+K_{M}*(1+[EP]/K_{I})\right)}$$

### Effect of ethyl pyruvate on human red blood cells

Heparinized human blood (5 ml) was centrifuged at 2000 rpm for 15 min and supernatant was removed. The cells were washed three times with 20 mM sodium phosphate, 150 mM sodium chloride (PBS), pH 7.4. Human red blood cells (5x10^5^) were mixed to 2x10^5^ *T*. *brucei* cells in 5 ml fresh medium in a 25 cm^2^ culture flask. Cells were incubated at 37°C and 5% CO_2_ for 24 hrs. Aliquots (1 ml) were taken and distributed into 24-well plates and incubated with 100 μl medium containing increasing concentration of ethyl pyruvate (1 to 15 mM). Controls contained cells without ethyl pyruvate but 100 μl of fresh medium. The cells were then incubated for 3 hrs. Trypanosome cells and blood cells were counted and the release of haemoglobin in the supernatant after cell centrifugation was measured photometrically at 540 nm. As a positive control a cell suspension of red blood cells was haemolysed using ultra-sonication to obtain complete hemolysis of a similar blood specimen not containing ethyl pyruvate.

### Phase contrast microscopy and video recording

Culture flasks containing 5 ml of cells (10^7^ cells/ml) were exposed to a final concentration of 5 mM ethyl pyruvate and 128 nM pentamidine. A control flask contained equal number of cells in fresh medium without trypanocidal compounds. All the cells were incubated for 3 hrs. For video recording, 5 ml cell suspension (10^7^ cells/ml) was poured into a Nunclon culture dish (Sigma-Aldrich, Taufkirchen, Germany) with a bottom partially replaced by a glass-slide to increase imaging quality. The dish was closed with a custom made lid equipped with a CO_2_ adapter to allow for 5% CO_2_ supply in air. Cells were observed for three hours on an inverted phase contrast microscope (DMIRB, Leica) endowed with a 20x Objective (0.4 NA) and a 1x C-mount. The microscope was enclosed by a self-made thermo-box kept at 37°C to provide usual cell culture conditions. Videos were recorded every hour for untreated and pentamidine treated cells. For those cells treated with 5 mM ethyl pyruvate video recording was done every 30 min. The recordings were taken using a Phytec FireWire-CAM-111H with a frame rate of 15 fps. Each video sequence lasts 2 min.

### Statistics

All analyses were done using GraphPad Prism 5.0 (GraphPad Software Inc. San Diego, USA). If not stated otherwise, all statistical tests were made by Mann-Whitney test. P<0.05 had been considered as significantly different.

## Supporting Information

S1 Video Phase contrast microscope video of live actively moving *T*. *brucei* cells. A control flask contained (10^7^ cells/ml) in 5 ml fresh medium without drugs and assigned as a negative control. The cells were incubated for 3 hrs and videos were recorded every hour (For details regarding the method please see the Materials and Methods section). A free downloadable version of Freemake Video Converter.exe software was used to sequentially put the video files together in one video file (Link: http://youtu.be/KIK_zZnJrCM) (login ID: netsanetworku; password: netsanet32000).
(DOCX)

S2 Video Phase contrast microscope video of pentamidine treated *T*. *brucei* cells. A test flask contained (10^7^ cells/ml) in 5 ml fresh medium treated with 128 nM pentamidine and treated as shown in S1 Video. (Link: http://youtu.be/xj5kKmWpz6o) (login ID: netsanetworku; password: netsanet32000).
(DOCX)

S3 Video Phase contrast microscope video of ethyl pyruvate treated *T*. *brucei* cells. A test flask contained (10^7^ cells/ml) in 5 ml fresh medium treated with 5 mM ethyl pyruvate and treated as shown in S1 Video (Link: http://youtu.be/xp72G_wZ8EU) (login ID: netsanetworku; password: netsanet32000).
(DOCX)
