# Supplementary material for: Ethyl Pyruvate Emerges as a Safe and Fast Acting Agent against Trypanosoma brucei by Targeting Pyruvate Kinase Activity
Source: PLoS One. 2015 Sep 4;10(9):e0137353. doi: 10.1371/journal.pone.0137353 (PMC4560413; doi:10.1371/journal.pone.0137353)
Supplement: S1 Video — A control flask contained (107 cells/ml) in 5 ml fresh medium without drugs and assigned as a negative control. The cells were incubated for 3 hrs and videos were recorded every hour (For details regarding the method please see the Materials and Methods section). A free downloadable version of Freemake Video Converter.exe software was used to sequentially put the video files together in one video file (Link: http://youtu.be/KIK_zZnJrCM) (login ID: netsanetworku; password: netsanet32000). (DOCX) [file pone.0137353.s001.docx]

**S1 Video**. **Phase contrast microscope video of live actively moving *T. brucei* cells S1 Video**. A control flask contained (10^7^ cells/ml) in 5 ml fresh medium without drugs and assigned as a negative control. The cells were incubated for 3 hrs and videos were recorded every hour (For details regarding the method please see the Materials and Methods section). A free downloadable version of Freemake Video Converter.exe software was used to sequentially put the video files together in one video file (Link: <http://youtu.be/KIK_zZnJrCM>) (login ID: netsanetworku; password: netsanet32000).
