# Supplementary material for: Ethyl Pyruvate Emerges as a Safe and Fast Acting Agent against Trypanosoma brucei by Targeting Pyruvate Kinase Activity
Source: PLoS One. 2015 Sep 4;10(9):e0137353. doi: 10.1371/journal.pone.0137353 (PMC4560413; doi:10.1371/journal.pone.0137353)
Supplement: S2 Video — A test flask contained (107 cells/ml) in 5 ml fresh medium treated with 128 nM pentamidine and treated as shown in S1 Video. (Link: http://youtu.be/xj5kKmWpz6o) (login ID: netsanetworku; password: netsanet32000). (DOCX) [file pone.0137353.s002.docx]

**S2 Video**. **Phase contrast microscope video of pentamidine treated *T. brucei* cells. S2 Video**. A test flask contained (10^7^ cells/ml) in 5 ml fresh medium treated with 128 nM pentamidine and treated as shown in Video S1. (Link: <http://youtu.be/xj5kKmWpz6o>) ((login ID: netsanetworku; password: netsanet32000)
