# Supplementary material for: Ethyl Pyruvate Emerges as a Safe and Fast Acting Agent against Trypanosoma brucei by Targeting Pyruvate Kinase Activity
Source: PLoS One. 2015 Sep 4;10(9):e0137353. doi: 10.1371/journal.pone.0137353 (PMC4560413; doi:10.1371/journal.pone.0137353)
Supplement: S3 Video — A test flask contained (107 cells/ml) in 5 ml fresh medium treated with 5 mM ethyl pyruvate and treated as shown in S1 Video (Link: http://youtu.be/xp72G_wZ8EU) (login ID: netsanetworku; password: netsanet32000). (DOCX) [file pone.0137353.s003.docx]

**S3 Video**. **Phase contrast microscope video of ethyl pyruvate treated *T. brucei* cells.** A test flask contained (10^7^ cells/ml) in 5 ml fresh medium treated with 5 mM ethyl pyruvate and treated as shown in Video S1 (Link: <http://youtu.be/xp72G_wZ8EU>) (login ID: netsanetworku; password: netsanet32000).
